# The role of the inspiratory muscle weakness in functional capacity in hemodialysis patients

**DOI:** 10.1371/journal.pone.0173159

**Published:** 2017-03-09

**Authors:** Pedro Henrique Scheidt Figueiredo, Márcia Maria Oliveira Lima, Henrique Silveira Costa, Rosalina Tossige Gomes, Camila Danielle Cunha Neves, Evandro Silveira de Oliveira, Frederico Lopes Alves, Vanessa Gomes Brandão Rodrigues, Emílio Henrique Barroso Maciel, Cláudio Heitor Balthazar

**Affiliations:** 1 Postgraduate Course in Physiological Sciences, Multicentric Program in Physiological Sciences, Universidade Federal dos Vales do Jequitinhonha e Mucuri, Diamantina, Minas Gerais, Brazil; 2 Cardiovascular Rehabilitation Laboratory (LABCAR), Physical Therapy School, Universidade Federal dos Vales do Jequitinhonha e Mucuri, Diamantina, Minas Gerais, Brazil; 3 Postgraduate Program in Infectious Diseases and Tropical Medicine, School of Medicine, Universidade Federal Minas Gerais, Belo Horizonte, Minas Gerais, Brazil; 4 Hemodialysis Unit of the Santa Casa de Caridade de Diamantina Hospital, Diamantina, Minas Gerais, Brazil; UFMG, BRAZIL

## Abstract

**Introduction:**

Inspiratory muscle function may be affected in patients with End-Stage Renal Disease (ESRD), further worsening the functional loss in these individuals. However, the impact of inspiratory muscle weakness (IMW) on the functional capacity (FC) of hemodialysis patients remains unknown. Thus, the present study aimed to evaluate the impact of IMW on FC in ESRD patients undergoing hemodialysis.

**Materials and methods:**

ESRD patients on hemodialysis treatment for more than six months were evaluated for inspiratory muscle strength and FC. Inspiratory muscle strength was evaluated based on maximal inspiratory pressure (MIP). IMW was defined as MIP values less than 70% of the predicted value. FC was evaluated using the Incremental Shuttle Walk test (ISWT). Patients whose predicted peak oxygen uptake (VO_2_peak) over the distance walked during the ISWT was less than 16mL/kg/min were considered to have FC impairment. Associations between variables were assessed by linear and logistic regression, with adjustment for age, sex, body mass index (BMI), presence of diabetes and hemoglobin level. Receiver-operating characteristic (ROC) analysis was used to determine different cutoff values of the MIP for normal inspiratory muscle strength and FC.

**Results:**

Sixty-five ERSD patients (67.7% male), aged 48.2 (44.5–51.9) years were evaluated. MIP was an independent predictor of the distance walked during the ISWT (R^2^ = 0.44). IMW was an independent predictor of VO_2_peak < 16mL/kg/min. (OR = 5.7; p = 0.048) in adjusted logistic regression models. ROC curves showed that the MIP cutoff value of 82cmH_2_O had a sensitivity of 73.5% and specificity of 93.7% in predicting normal inspiratory strength and a sensitivity and specificity of 76.3% and 70.4%, respectively, in predicting VO_2_peak ≥ 16mL/kg/min.

**Conclusions:**

IMW is associated with reduced FC in hemodialysis patients. Evaluation of the MIP may be important to functional monitoring in clinical practice and can help in the stratification of patients eligible to perform exercise testing.

## Introduction

Chronic kidney disease is a worldwide public health problem, leading to irreversible functional impairment of the kidneys in affected patients [1]. In patients with End-Stage Renal Disease (ESRD), both kidney disease and hemodialysis treatment may severely affect the cardiorespiratory and musculoskeletal systems, contributing to a reduction in functional capacity (FC) [2, 3]. Functional impairment adversely impacts patients’ physical and mental capabilities and, consequently, their ability to perform daily activities and maintain independence and social interactions [4–7]. Furthermore, decreased FC is one of the major risk factors for cardiac death in this population [8–10], which is 10 to 20 times higher than in the general population [11]. Given the association between FC, adverse cardiovascular events and quality of life, the evaluation and identification of risk factors for FC impairment is highly relevant to ESRD management [12].

Inspiratory muscle weakness (IMW), as determined by maximal inspiratory pressure (MIP), is associated with FC in some populations that suffer from muscle wasting [13, 14]. In ESRD, uremic myopathy may influence the loss of strength and endurance in inspiratory muscles, as well as in muscles of the locomotor system [15]. In fact, some studies have found a decreased MIP in hemodialysis patients when compared to healthy individuals and a correlation between MIP and FC [16, 17]. However, the effect of MIP on FC, the association between IMW and low FC, and values of MIP that predict IMW and low FC in hemodialysis patients remain unknown. Therefore, the aim of the present study was to evaluate the effect of IMW on the FC of ESRD patients. Clarifying these questions may contribute to the functional monitoring of ESRD patients and the development of prevention and treatment strategies for them.

## Materials and methods

### Study design

This cross-sectional study was conducted in the hemodialysis unit of the Santa Casa de Caridade de Diamantina Hospital and the Cardiovascular Rehabilitation Laboratory (LABCAR) of the Universidade Federal dos Vales Jequitinhonha e Mucuri (Diamantina-Minas Gerais state, Brazil). The research was carried out in accordance with the declaration of Helsinki [18] and was approved by ethics committee of the Universidade Federal dos Vales do Jequitinhonha e Mucuri (protocol 088/12). All the patients gave their written informed consent before participating in the study.

ESRD patients older than 18 years who were receiving hemodialysis treatment thrice weekly for at least six months between May 2013 and October 2014 and had an arteriovenous fistula for hemodialysis access were included in the study. Exclusion criteria were contraindications or inability to perform the functional tests.

The selected patients underwent clinical evaluation by nephrologists following a structured anamnesis protocol. Anthropometric measurements (weight, height, body mass index (BMI) and waist circumference), FC, inspiratory muscle strength and blood biochemistry of the volunteers were also evaluated. The investigators were blinded to test results, and all volunteers had previously been trained to perform the functional tests.

All evaluations were performed on dialysis days, immediately before hemodialysis sessions.

### Functional capacity

FC was evaluated using the Incremental Shuttle Walk Test (ISWT), according to the protocol proposed by Singh et al. [19]. Volunteers were instructed to walk or run in a 10 m corridor and the minimum speed was determined by an audio signal. The ISWT has 12 progressive intensity levels, and the test is completed when the volunteer either completes the 12 levels of intensity or fails to reach the minimum speed required on a given level two consecutive times [19]. Vital signs were monitored during the test, and the distance walked was recorded. Results are recorded both as absolute values and as relative values based on the percentage achieved compared to the maximum predicted [20]. For the purposes of this analysis, we defined FC impairment based on a predicted peak oxygen uptake (VO_2_peak) less than 16 mL/kg/min [21] over the distance walked during the ISWT [20].

### Inspiratory muscle strength

Respiratory muscle strength was determined using a previously calibrated aneroid vacuum manometer (MV-150/300, Ger-Ar, São Paulo, Brazil) equipped with a 2 mm diameter hole in the nozzle to compensate for the pressure change induced by the oropharynx muscles. MIP was evaluated based on residual volume while the volunteers were seated, and the highest value of three valid measurements was retained [22]. The measurements were considered satisfactory if variance between them was at most 10%. Respiratory measurements are shown as both absolute and relative values based on the percentage achieved compared to the maximum predicted by age and sex [23]. IMW was defined as MIP less than 70% of the predicted value [22].

### Laboratory analysis

Ethylenediaminetetraacetic acid and clot activator vacutainers (BD vacutainers, Franklin Lakes, NJ, USA) were used to collect 10mL venous blood samples immediately before the second hemodialysis session of the week (midweek). The tubes were centrifuged at 2500 rpm for 10 min at 4°C, and aliquots of plasma and serum (500 μL) were stored in a freezer at -80°C for biochemical analysis using standard laboratory techniques.

### Statistical analysis

Statistical analysis was performed using SPSS version 22.0 (SPSS Inc., Chicago, IL, USA). The data distributions were verified using the Kolmogorov-Smirnov test, and outliers were detected using boxplots. Categorical variables are presented as absolute and relative frequencies, and continuous variables are presented as the mean (95% confidence interval (CI)). Correlation analysis was carried out using the Pearson or Spearman tests (continuous variables) or the Chi-squared test (categorical variables).

The association between MIP and FC was assessed by univariate analysis, followed by stepwise multivariate linear regression analysis, with adjustment for age, sex, BMI, hemoglobin (Hb) and presence of diabetes. Due to non-homogeneity of variance observed in residual analysis (Durbin-Watson), a logarithmic transformation of the dependent variable (ISWT distance) was performed prior to regression analysis.

Univariate logistic regression analysis was performed to evaluate the association between IMW and FC. If p<0.1, multivariate analysis was performed to evaluated the odds of individuals with IMW having VO_2_peak less than 16 mL/kg/min, after adjusting for age, sex, BMI, Hb and presence of diabetes. The significance level in the multivariate analysis was set at 0.05.

Receiver-operating characteristic (ROC) analysis was performed to determine the sensitivity and specificity of different cutoff values of the MIP for the prediction of normal inspiratory muscle strength. ROC analysis was also used to determine cutoff values of the MIP and percentage of the prediction of MIP in identifying FC ≥ 16 mL/kg/min. The ideal cutoff points were defined by values with the best combination of sensitivity and specificity.

## Results

Out of a total of 93 ESRD patients, 78 were selected and 65 volunteers were enrolled in the study (one did not provide consent, two had severe anemia, two had complex cardiac arrhythmias, one had angina, five had lower limb disability and two did not perform all the steps of the evaluation protocol). Descriptive characteristics of all the study participants are presented in Table 1. The volunteers were predominantly male (67.7%), with a mean age of 48.2 (44.5–51.9) years, and eutrophic. Systemic arterial hypertension was the most prevalent etiology of ESRD. All volunteers were taking vitamin C and B complex, and 59 (90.8%) were using erythropoietin. Dialysis data (kt/v indexes and urea reduction rate) demonstrated the efficiency of hemodialysis treatment.

**Table 1:** Characteristics of the sample.

	n = 65
**Sex**	
Male	44 (67.7)
Female	21 (32.3)
**Race**	
Caucasian	22 (33.8)
Black	23 (35.4)
Other	20 (30.8)
**Age (years)**	48.2 (44.5–51.9)
**Dry weight (kg)**	59.9 (54.5–65.2)
**Height (m)**	1.6 (1.6–1.7)
**BMI (kg/m**^**2**^**)**	24.1 (22.9–25.4)
**Duration of hemodialysis (years)**	4.8 (3.7–5.8)
**ESRD etiology**	
Hypertensive nephropathy	19 (29.2)
Diabetic nephropathy	13 (20.0)
Glomerulonephritis	9 (13.9)
Polycystic kidneys	2 (3.1)
Others	22 (33.8)
**Comorbity condictions**	
Systemic Hypertension	54 (83.1)
Diabetic	15 (23.1)
Obesidity	7 (10.8)
Tabagism	9 (13.8)
**Antihypertensive medications**	
β-blockers	49 (75.4)
Calcium Antaginist	15 (23.0)
Angiotensin II receptor antagonist	14 (21.5)
Diuretic	33 (50.8)
ACE-inhibitor	9 (18.8)
**Biochemist**	
Kt/V	1.6 (1.5–1.6)
Urea (mg/dl)	152.0 (141.5–162.4)
Urea reduction ratio, (%)	71.7(70.2–73.2)
Albumin (g/dL)	3.8 (3.7–4.0)
Hemoglobin (mg/dL)	10.8 (10.2–11.2)
Hematocrit (%)	33.9 (32.7–35.0)
**MIP (cmH**_**2**_**O)**	89.5 (82.9–96.1)
**Predicted MIP (%)**	84.0 (79.0–89.2)
**ISWT**	
Distance (m)	416.5 (360.9–472.0)
Predicted distance (%)	67.4 (59.8–74.9)
Predicted VO_2_peak (mL/kg/min)	18.9 (16.8–21.0)

Data represented as mean (IC 95%) or n(%). BMI: body mass index; ESRD: End-Stage Kidney Disease; ACE: angiotensin converting enzyme. Kt/V: dialysis efficiency; MIP: maximal inspiratory pressure; ISWT: incremental shuttle walk test; VO_2_peak: peak oxygen uptake.

IMW was observed in 16 patients (24.6%). However, in the overall study population, there was no clinically significant reduction in mean MIP compared with predicted values. FC impairment (VO_2_peak < 16 mL/kg/min) was observed in 27 volunteers (41.5%).

There was a significant correlation between MIP and FC (r = 0.48; p<0.001). The results of the linear regression analysis are presented in Table 2. Higher MIP was associated with significantly higher FC (*b* = 0.005; p < 0.001), with an adjusted R^2^ of 0.26. In the multivariate analysis, MIP was an independent predictor of FC. MIP and age explained 40% of changes in FC (*b* = 0.003; p = 0.001 and *b* = -0.007; p < 0.019, respectively). When diabetes was included, higher MIP (*b* = 0.004; p = 0.012), lower age (*b* = -0.007; p <0.001) and absence of diabetes (*b* = 0.145; p = 0.017) were significantly associated with FC (adjusted R^2^ = 0.44). Age was the strongest independent predictor of FC in multivariate analysis (adjusted R^2^ = 0.30). The residual analysis showed a normal distribution and homogenous variance in all the regression models.

**Table 2:** Results of the linear regression analysis.

	Predictors	R^2^ adjusted	*b* ± SE	β	p
**Model: MIP**					
	MIP	0.26	0.005 ± 0.001	0.516	<0.001
**Model: MIP, age, sex BMI, diabetes and Hb**					
1	Age	0.30	-0.010 ± 0.002	-0.557	<0.001
2	Age	0.40	-0.007 ± 0.002	-0.420	<0.001
	MIP		0.003 ± 0.001	0.355	0.001
3	Age	0.44	-0.007 ± 0.002	-0.302	<0.001
	MIP		0.004 ± 0.001	0.460	0.012
	Diabetes		0.145 ± 0.059	0.231	0.017

MIP: maximal inspiratory pressure; Dependent variable: distance of incremental shuttle walk test (Log scale).

IMW was associated with FC, as demonstrated by the chi-squared test (χ2 = 6.45; p = 0.011). The results of the logistic regression analysis are presented in Table 3. In the univariate logistic regression analysis, the presence of IMW predicted VO_2_peak < 16 mL/kg/min, with an odds ratio of 4.5 (1.4–15.3). In the multivariate analysis, IMW was an independent predictor of VO_2_peak < 16 mL/kg/min, after adjusting for age, sex, BMI, Hb and presence of diabetes (odds ratio = 5.7 (1.1–31.4); p = 0.048)). Age and diabetes were other independent predictors in the model.

**Table 3:** Results of the logistic regression analysis.

Variables	*b* ± SE	OR	IC95%	p
**Model 1 –IMW**				
IMW	2.512 ± 0.619	4.5	1.4–15.3	0.015
**Modelo 2—IMW, age, sex, diabetes and Hb**				
IMW	1.731 ± 0.875	5.7	1.1–31.4	0.048
Age	-0.118 ± 0.033	0.9	0.8–0.9	0.001
Diabetes	2.042 ± 0.870	7.7	1.4–42.4	0.019
BMI				NS
Sex				NS
Hb				NS

IMW: inspiratory muscle weakness; BMI: body index mass; Hb: hemoglobin; Dependent variable: impairment of the functional capacity by distance of incremental shuttle walk test (VO_2_peak <16mL/kg/min).

**Fig 1:**
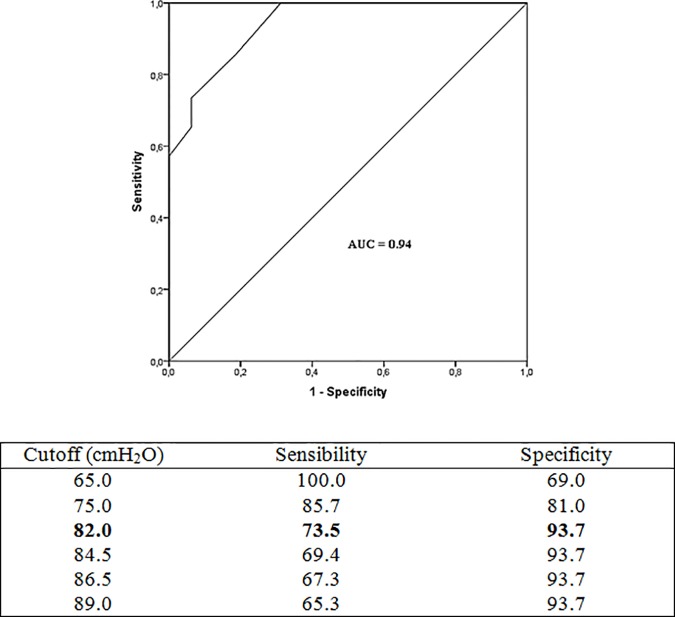
Cutoff of Maximal Inspiratory Pressure to normal inspiratory strength. MIP: maximal inspiratory pressure. Normal inspiratory strength: MIP ≥ 70% predict.

Results of the ROC analysis (MIP vs. IMW) are shown in Fig 1. The area under the curve for this analysis was 0.94. The MIP cutoff of 82 cmH_2_O had a sensitivity of 73.5% and a specificity of 93.7% in identifying normal inspiratory strength (MIP ≥ 70% of predicted value). In this analysis, higher MIP cutoffs yielded lower sensitivity, while specificity remained the same.

**Fig 2:**
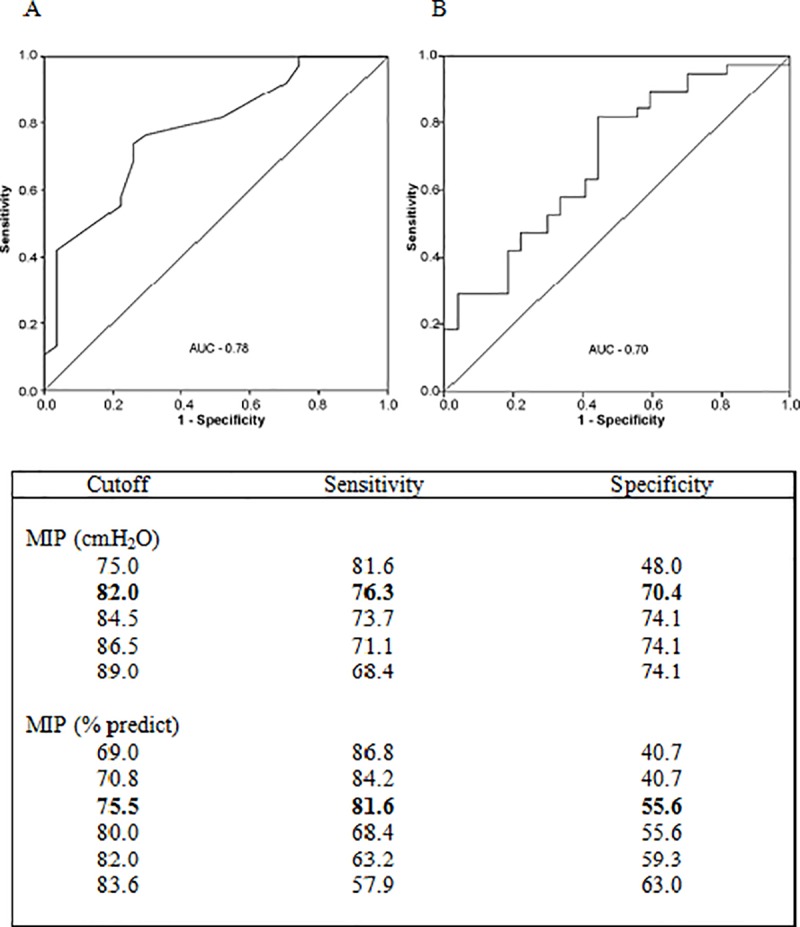
Cutoff of MIP and percentage of predict MIP to normal functional capacity. MIP: maximal inspiratory pressure. A: MIP vs normal functional capacity; B: % of predict MIP vs normal functional capacity. Normal functional capacity was considered when VO_2_peak in incremental shuttle walk test ≥ 16 mL/kg/min.

Fig 2 shows the results of the ROC analyses of MIP and percentage predicted MIP vs. VO_2_peak. The areas under the curve for these analyses were 0.79 and 0.70, respectively. The MIP cutoff of 82 cmH_2_O of had a sensitivity of 76.3% and a specificity of 70.4% in predicting VO_2_peak ≥ 16 mL/kg/min, and the cutoff of 75.5% predicted MIP had a sensitivity of 81.6% and specificity of 55.6%.

## Discussion

To the best of our knowledge, this is the first study to demonstrate that FC is influened by inspiratory muscle strength, even when other factors known to be associated with physical capacity were considered. The main findings of the present study were (1) the high prevalence of IMW in ERSD patients, (2) the association between IMW and FC and (3) MIP cutoff values to identify patients with FC impairment. These results have important clinical significance, as MIP is an inexpensive and easy-to-use method that can aid in the development of functional prevention and treatment strategies for this population.

In our first analysis, we evaluated the association between MIP and FC. The results showed that MIP explains 26% of variation in the distance walked during the ISWT (log scale). In the adjusted model, MIP was an independent predictor of FC. MIP, age and presence of diabetes explained 44% of variation in FC. The results of our second analysis (logistic regression) showed that IMW is significantly associated with VO_2_peak < 16 mL/kg/min, with an odds ratio of 4.5 (1.4–15.3) in univariate analysis. In the adjusted model, IMW increased the odds of VO_2_peak < 16 mL/kg/min by 5.7 times independent of age, diabetes, BMI, sex and hemoglobin level (OR 5.7 (1.1–31.4)). Age and presence of diabetes were other predictors of reduced FC. The influence of age and diabetes on the clinical and functional status of hemodialysis patients has been previously described [9, 24–26].

The association between MIP and FC has already been described in other health conditions [13, 14, 27–29]. In hemodialysis patients, the association between these variables has only been previously demonstrated by correlation analysis [16, 17, 30, 31]. This made it difficult to compare our results with other findings. The mechanisms involved in this association are unclear, but there may be factors that are associated with MIP and also have an impact on functional impairment in ESRD. Both uremic myopathy and hemodialysis promote protein breakdown [15], which may affect the strength and endurance of both inspiratory muscles and peripheral muscles. Thus, reduced muscle oxidative capacity, protein energy wasting [32], vitamin D deficiency [33], elevated inflammatory response [34, 35] and volemic status [36] are common findings that are possibly associated with reduced MIP. Once MIP is compromised, performance in activities requiring increased metabolic demand becomes limited [37]. Additionally, reduced MIP may contribute to the early use of accessory inspiratory muscles during effort. This compensatory mechanism is associated with dynamic distortions of the chest wall and, consequently, reductions in ventilatory efficiency [38].

Another potential mechanism associated with IMW-induced functional impairment is cardiorespiratory interaction. In subjects with IMW, the increased respiratory effort during exercise may increase metabolic stress in inspiratory muscles and local metaboreflex stimulus. Therefore, there may be increased blood flow to inspiratory muscles with simultaneous reduction in perfusion to locomotor muscles, resulting in early fatigue [39], as has been found in heart failure patients [40]. However, this outcome has not been assessed in ESRD patients undergoing hemodialysis.

Given that reduced FC is a traditional cardiovascular risk factor and a well-established independent predictor of mortality in ESRD [8–10], the convenience of evaluation of MIP and the associations found herein, we investigated cutoff MIP values that can identify normal inspiratory muscle strength and VO_2_peak ≥ 16 mL/kg/min using ROC analysis. MIP values higher than 82 cmH_2_O predicted normal inspiratory muscle strength with a sensitivity of 73.5% and 6.3% false positives (specificity = 93.7%), independent of sex and age. Thus, an MIP of 82 cmH_2_O is a good cutoff for stratification of hemodialysis patients with or without IMW. This value is close to the previously proposed level of 80 cmH_2_O, which is used in excluding clinically significant IMW in the general population [22].

The same MIP cutoff value predicted VO_2_peak ≥ 16 mL/kg/min with a sensitivity and specificity of 76.3% and 70.4%, respectively. These results show that an MIP of 82 cmH_2_O may be used in stratification of patients with or without FC impairment, with moderate sensitivity and specificity. A cutoff of 75.5% predicted MIP (determined by age and sex) was sensitive but not specific (sensitivity = 81.6% and specificity = 55.6%) in identifying patients with VO_2_peak ≥ 16 mL/kg/min. This points to the low probability of normal FC in patients with MIP less than 75.5% of the predicted value but does not allow exclusion of reduced FC in patients with MIP above this cutoff, suggesting a need for additional tests such as exercise testing.

Put together, the findings of this study suggest that reductions in MIP contribute to decreased FC. However, IMW increases the chance of reduced FC by more than 5-fold. In addition, MIP measurement is easy and inexpensive, has few contraindications, does not require much space, and may aid in the stratification of patients eligible to perform functional assessment by exercise testing as well as in evaluating the effects of interventions on functional status. The inclusion of MIP evaluation in functional assessment protocols for hemodialysis patients may be clinically relevant, as IMW is an independent risk factor for myocardial infarction (HR 1.48) and cardiovascular death (HR 1.54) in the general population [41] and has been associated with autonomic dysfunction in cardiovascular [42, 43] and pulmonary diseases [44]. It is important to note that the relationships between IMW and clinical parameters in ESRD, such as mortality and morbidity, should be further investigated.

The present study has some limitations. The criteria for dichotomization of FC were based on VO_2_ (16 mL/kg/min) according to the Weber classification [21] and obtained by indirect measurement. However, given that ISWT is a functional test with high correlation with the cardiopulmonary exercise test [12, 45] and that individuals can reach VO_2_peak during ISWT similar to the maximal effort test [46–48], we believe that this limitation did not influence the findings.

In conclusion, our study shows that IMW is associated with reduced FC in hemodialysis patients. Additionally, in clinical practice, the evaluation of MIP may be a useful method for functional assessment and monitoring and evaluation of interventions and can aid in stratifying of patients eligible to perform functional assessment by effort tests. Studies to evaluate the influence of IMW on clinical parameters in this population should be performed.
